# CAMKK1 in Obesity and Type 2 Diabetes Mellitus: Evidence of Interaction With Appetite‐Regulating, Metabolic and Inflammatory Factors

**DOI:** 10.1002/edm2.70109

**Published:** 2025-09-18

**Authors:** Livio Tarchi, Lorenzo Bonacchi, Andrea Di Santo, Paolo Rovero, Chiara Sassoli, Rachele Garella, Roberta Squecco, Gianluca Villa, Romina Nassini, Francesco De Logu, Valdo Ricca, Giovanni Castellini

**Affiliations:** ^1^ Department of Health Sciences—Center for Integrated Translational Research and Knowledge Transfer (INCLINE) University of Florence Florence Italy; ^2^ Department of Neuroscience, Psychology, Pharmacology and Infant Health University of Florence Florence Italy; ^3^ Department of Experimental, Clinical, and Biomedical Sciences “Mario Serio” University of Florence Florence Italy

**Keywords:** calcium/calmodulin‐dependent protein kinase, energy homeostasis, inflammation, metabolic regulation, overweight

## Abstract

**Introduction:**

Calcium/calmodulin‐dependent protein kinase kinase 1 (CAMKK1) regulates energy homeostasis through AMP‐activated protein kinase (AMPK). CAMKK1 has been implicated in appetite and satiety regulation; however, its role in obesity or type 2 diabetes mellitus (T2DM) remains unexplored. In this cross‐sectional study, the primary aim was to confirm whether CAMKK1 is elevated in individuals with diabetes. The secondary aim was to investigate CAMKK1's molecular correlates.

**Methods:**

CAMKK1 serum levels in individuals with obesity (*n* = 3,061), patients with T2DM (*n* = 4,910) and controls (*n* = 44,257) were retrieved and compared (age, body mass index—BMI and sex‐adjusted ANCOVA). Pearson correlation coefficients and linear regression coefficients (age and BMI‐adjusted) were computed. The moderation effect of diagnostic groups was also assessed. The interaction between factors was explored by mixed graphical models.

**Results:**

CAMKK1 was elevated in patients with T2DM, in comparison to both individuals with obesity and controls (post hoc comparison, Tukey‐adjusted *p* = 0.010 and *p* = 0.044, respectively). Across diagnostic groups, positive associations were observed between CAMKK1 and AMPK (min *β* > 0.400, max *p* < 0.001) or TNFα (min *> β* 0.070, max *p* < 0.001). A positive association with leptin (*β* = 0.010, *p =* 0.002) and ghrelin (*β =* 0.005, *p =* 0.048) was observed only within controls. Multivariate multivariable models confirmed that specific interactions between factors were disrupted in patients with T2DM (*p* < 0.001).

**Conclusion:**

These findings provide new insights into the role of CAMKK1 in obesity and T2DM. Future research may further explore CAMKK1's interplay with inflammatory pathways.

## Graphical abstract

Elevated CAMKK1 levels were observed in patients with T2DM, in comparison to both individuals with obesity and controls. In the whole cohort, higher CAMKK1 was associated with higher AMPK and TNFα. The interactions between serum factors were disrupted in patients with T2DM.

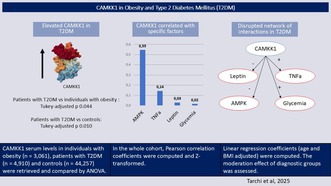

## 1 Introduction

Obesity and diabetes are chronic conditions, significantly contributing to the overall burden of disease at the individual and global level [1, 2]. Obesity and diabetes share common risk factors, including metabolic, inflammatory and appetite‐regulating hormones [3, 4]. Recent estimates describe up to 52% of the health burden of diabetes to actually be attributable to obesity [1], and recent forecasts predict both obesity and diabetes to continue increasing in prevalence at least up to 2050 [1, 2].

While in recent decades effective treatments for diabetes and weight‐loss have been developed [5], individuals with obesity or patients with diabetes still report several neglected needs. Among these, current research has highlighted the demand for individualised algorithms, capable of identifying diabetes or pre‐diabetes at an earlier stage of development [6] and thus possibly preventing the onset of the disease. For this aim, a better characterisation of the calcium/calmodulin‐dependent signalling pathway (including kinases, CAMKs) offers promising potential for future research and clinical practice, as this pathway exhibits notable cross‐talks with appetite regulation [7], metabolic [8] and inflammatory hormones [9].

Evidence has been consolidating on the role of specific CAMKs variants in both obesity [10] and diabetes [11], either through hypomethylation [12] or increased recognition of transcription factors [13]. Notably, previous tissue studies have also shown increased CAMKs expression in human islet cells of diabetic patients in comparison to controls [14]. However, to the authors' knowledge, the full translation of these findings has yet to be described, even though the interplay between CAMKs, AMPK and appetite‐regulating hormones (i.e., promoting ghrelin‐mediated food intake, and possibly reducing leptin‐mediated satiety) may be of specific interest to obesity and diabetes research [15].

Within CAMKs' pathways, a specific kinase, calcium/calmodulin‐dependent protein kinase kinase 1 (CAMKK1), has recently gathered interest for its role in orchestrating different signal cascades [16]. In fact, CAMKK1 plays a crucial role in activating other CAMKs and several other signalling pathways [17]. CAMKK1 is known to regulate energy homeostasis through the phosphorylation of AMP‐activated protein kinases (AMPK) [17]. CAMKK1's downstream pathway also includes general transcription activation (e.g., CREB, through CAMKs), neuronal development [18] and cell survival [19]. However, similarly to other CAMKs, the potential interplay between CAMKK1, metabolic and inflammatory factors has not yet been fully described.

Nonetheless, inflammatory cytokines have long emerged as a crucial factor in relation to the risk of development or progression of metabolic dysregulation, in either obesity [20] or diabetes [21]. For this reason, in light of previous evidence on the regulatory role of CAMKK1 on neutrophil differentiation and functional activation [22], and in light of the role of other CAMKs in cancer immune resistance [23], it is reasonable to posit that CAMKK1 may also play a role in the cross‐talk between metabolic and inflammatory factors [24, 25, 26].

On these premises, the current work aimed at: (i) confirming whether CAMKK1 is elevated in individuals with obesity and patients affected by diabetes, hypothesising that patients with diabetes would exhibit elevated CAMKK1 levels in comparison to both patients with obesity and controls; (ii) confirming whether CAMKK1 is associated with appetite‐regulating factors (i.e., positively with satiety promoting factors—leptin, negatively with factors promoting food intake—ghrelin); (iii) confirming whether CAMKK1 serum levels are positively associated with metabolic (i.e., AMPK, glycemia) and inflammatory factors (i.e., Tissue Necrosis Factor alpha—TNFα); (iv) confirming whether the cross‐talk between CAMKK1 and other hormones is perturbated in type 2 diabetes mellitus (T2DM).

## 2 Materials and Methods

### 2.1 Sample

First, the UKBiobank database was filtered to retain only participants with a diagnosis of obesity (*n* = 3,061) or type 2 diabetes mellitus (*n* = 4,910; T2DM). Further information on UKBiobank sampling methods, or specific information on protein level assessment, may be retrieved in the parent study [27]. Diagnoses were encoded in categorical features, and if a participant with T2DM also had obesity, the diagnosis was encoded as T2DM. A total of 1264 individuals had both obesity and T2DM (25.74% of the overall sample of patients with T2DM), a rate similar to that described by epidemiological studies [28]. For the group of controls, individuals with a lifetime history of obesity, T2DM, or an eating disorder (anorexia nervosa, bulimia nervosa, any other eating disorder) were excluded (final included sample, *n* = 44,257). Blood glucose (glycemia) was retrieved for all included participants.

### 2.2 OLINK Proteomics

Of the initial ~500,000 UK Biobank participants, a subset was selected for further analyses of their plasma samples using the OLINK Explore 1536 Proteomics platform, relying on samples collected at baseline assessment. Normalised protein expression (NPX) through Log2 transformation is used to represent plasma protein data. The following proteins were retrieved in the present study: CAMKK1, ghrelin, leptin, TNFα, AMPK (i.e., PRAKB1.5). For patients with T2DM, serum levels of glycated haemoglobin (HBA1c) were also retrieved, as well as diagnostic codes indicative of clinical complications due to diabetes (any or any combination of the following diagnoses: diabetic mononeuropathy, diabetic polyneuropathy, diabetic cataract, diabetic retinopathy, diabetic arthropathy). For more details on OLINK proteomics, see the parent study [29].

### 2.3 Ethics Approval

The study was approved by the North West Centre for Research Ethics Committee (11/NW/0382), and the participants provided signed consent before examination. See the parent study for further information [27].

### 2.4 Statistical Methods

First, descriptive statistics were computed for each diagnostic category (controls, individuals with obesity, patients with T2DM), including the number of missing values, the mean and the standard deviation. Age and body mass index (BMI) differences between groups were estimated by analysis of variance (ANOVA). Differences in sex ratios between groups were estimated by Chi‐squared test. Protein levels (CAMKK1, leptin, ghrelin TNFα, AMPK) and glycemia were compared between each diagnostic group by analysis of covariance (ANCOVA), adjusted for age, body mass index (BMI) and sex.

Correlation coefficients (Pearson's *r*, age and BMI‐adjusted) were estimated between CAMKK1 and appetite‐regulating (i.e., leptin, ghrelin), metabolic (i.e., AMPK and glycemia), or inflammatory factors (i.e., TNFα). Effect sizes were computed by Fisher's *z*. These associations were then tested by multivariable regression, within each diagnostic group, with CAMKK1 as the dependent variable and leptin, ghrelin, AMPK, glycemia and TNFα as independent variables. All variables were adjusted for age, sex and BMI as independent variables.

Then, a multivariable linear model assessed the hypothesis of diverging effects within diagnostic groups. The moderating role of diagnostic groups in the relationship between CAMKK1 and leptin, ghrelin, AMPK, glycemia and TNFα was investigated by linear regression. Each factor represented the dependent variable, while CAMMK1, age, sex, diagnostic group and BMI were the independent variables. To assess moderating effects, an interaction term between CAMKK1 and diagnostic groups was also computed. Clinical associations, for patients with T2DM alone, were tested by multivariable regression, with CAMKK1 as the dependent variable and glycated haemoglobin (HBA1c) as the independent variable. All variables were adjusted for age, sex and BMI as independent variables. Potential differences between patients treated and not treated with either (1) insulin products, (2) metformin and (3) both drugs, were tested by ANCOVA, adjusted for age, BMI and sex. Clinical comorbidities were tested by multivariate logistic regression, with complications due to diabetes as the dependent variable and CAMKK1, age, BMI and sex as independent variables. As individuals with diabetic complications were posited to be more likely to undergo diabetic treatment, treatment with insulin products or metformin was also included as independent variables. Interaction factors between CAMKK1 and sex, CAMMK1 and age, CAMMK1 and BMI were computed.

Subsequently, a network analysis was carried forward. In brief, network analyses can capture the higher dimensional structure between different factors, as well as potential differences between groups (in relation to both structure and strength of associations). The nodes (factors) and edges (links between nodes) of the network were estimated through a mixed graphical model after a 10‐fold cross‐validation [30], both before (overall) and after dividing the sample according to diagnostic groups. In summary, mixed graphical models are a generalisation of Gaussian models [31], and mixed graphical models allow for the estimation of regularised node‐wise regressions (pairwise association between each node, after regularisation by group‐LASSO) [31].

The potential difference between networks computed across different diagnostic groups was estimated by *NetworkComparisonTest*. As a single network estimation might not be fully representative of the underlying population, edge weights were compared between networks after performing bootstrapping (*N* = 5,000) [32]. The empirical *p*‐value obtained through permutation testing was then corrected for False Discovery Rate (FDR), as calculated by the Benjamini & Hochberg procedure [33].

All analyses were performed using JASP 0.19.3 [34] and R 4.3.3 [35], with the support of the *tidyverse* collection of libraries for data management [36], *bootnet* for network estimation [37], *NetworkComparisonTest* [32].

## 3 Results

An overall sample of 52,228 individuals was selected (individuals with obesity, *n* = 3,061; patients with T2DM, *n* = 4,910; controls, *n* = 44,257). On average, participants were 56.82 years old (standard deviation = 8.21), and mostly female (28,138 females vs. 24,090 males; 53.88% and 46.13% of the sample, respectively). Included patients with T2DM were mostly within acceptable glycemic control (mean HBA1c 47.82 ± 13.83, missing *n* = 247, 5%; below 48 mmol/mol *n* = 3005, 61.2%; between 48 and 53 mmol/mol *n* = 529, 10.8%; between 53 and 58 mmol/mol *n* = 362, 7.4%; above 58 mmol/mol *n* = 767, 15.6%). Most included patients with T2DM were not under active pharmacological treatment (any insulin product *n* = 490, 10%; metformin *n* = 1,443, 29.4%; both insulin and metformin *n* = 247, 5%). A minority of the sample of patients with T2DM had complications due to diabetes (any of diabetic mononeuropathy, polyneuropathy, cataract, retinopathy, arthropathy; *n* = 662, 13.5%). Further descriptive statistics were reported in Table 1.

**TABLE 1:** Sample descriptives.

	Controls	Obesity	Type 2 diabetes mellitus	*F*‐value/chi‐squared	Post hoc comparisons
*N*	44,257	3,061	4,910	—	—
Sex^a^	Male: 19,856 Female: 24,401	Male: 1,300 Female: 1,761	Male: 2,934 Female: 1,976	411.826***	—
Age^b^	56.43 ± 8.25 (missing = 0)	57.37 ± 7.87 (missing = 0)	59.96 ± 4.14 (missing = 0)	421.643***	Controls vs. Obesity*** Obesity vs. T2DM*** Controls vs. T2DM***
BMI^b^	26.65 ± 4.14 (missing = 181)	33.03 ± 5.13 (missing = 20)	31.46 ± 5.61 (missing = 50)	5.260***	Controls vs. Obesity*** Obesity vs. T2DM*** Controls vs. T2DM***
CAMKK1	0.04 ± 0.50 (missing = 2,206)	0.07 ± 0.49 (missing = 158)	0.09 ± 0.49 (missing = 284)	4.728**	Controls vs. Obesity: N.S. Obesity vs. T2DM** Controls vs. T2DM*
Leptin	−0.21 ± 1.28 (missing = 1,217)	0.85 ± 1.23 (missing = 88)	0.47 ± 1.19 (missing = 144)	21.087***	Controls vs. Obesity*** Obesity vs. T2DM*** Controls vs. T2DM**
Ghrelin	0.01 ± 0.96 (missing = 1,217)	−0.21 ± 0.98 (missing = 88)	−0.46 ± 1.04 (missing = 144)	112.272***	Controls vs. Obesity* Obesity vs. T2DM*** Controls vs. T2DM***
AMPK	0.06 ± 0.61 (missing = 2,006)	0.12 ± 0.59 (missing = 146)	0.14 ± 0.60 (missing = 256)	4.244***	Controls vs. Obesity: N.S. Obesity vs. T2DM: N.S. Controls vs. T2DM**
Glycemia	4.98 ± 0.76 (missing = 5,307)	5.01 ± 0.67 (missing = 358)	6.65 ± 3.00 (missing = 564)	3,211.218***	Controls vs. Obesity*** Obesity vs. T2DM*** Controls vs. T2DM***
TNFα	0.01 ± 0.43 (missing = 2,073)	0.11 ± 0.43 (missing = 151)	0.19 ± 0.45 (missing = 238)	100.090***	Controls vs. Obesity: N.S. Obesity vs. T2DM*** Controls vs. T2DM***

*Note:* Post hoc comparison by mean difference, post hoc *p*‐value adjusted by Tukey. Group differences corrected for age, sex and BMI. ± = standard deviation. N.S. = *p* > 0.05, ***p < 0.001, **p < 0.01, *p < 0.5.

Abbreviations: BMI, body mass index; T2DM, type 2 diabetes.

^a^ Chi‐squared test.

^b^ Not adjusted for age or BMI.

### 3.1 Group Differences

While sex differences were observed in controls (females exhibited lower CAMKK1s in comparison to males, post hoc Tukey‐adjusted *p* < 0.001; ANCOVA age, BMI and sex‐adjusted, with an interaction term between sex and diagnostic group), no difference was observed between males and females for individuals with obesity (Tukey‐adjusted *p* = 0.397) nor patients with T2DM (Tukey‐adjusted *p* = 0.512). See Table S1 for further results of post hoc comparisons, within diagnostic groups, accounting for sex.

**FIGURE 1:**
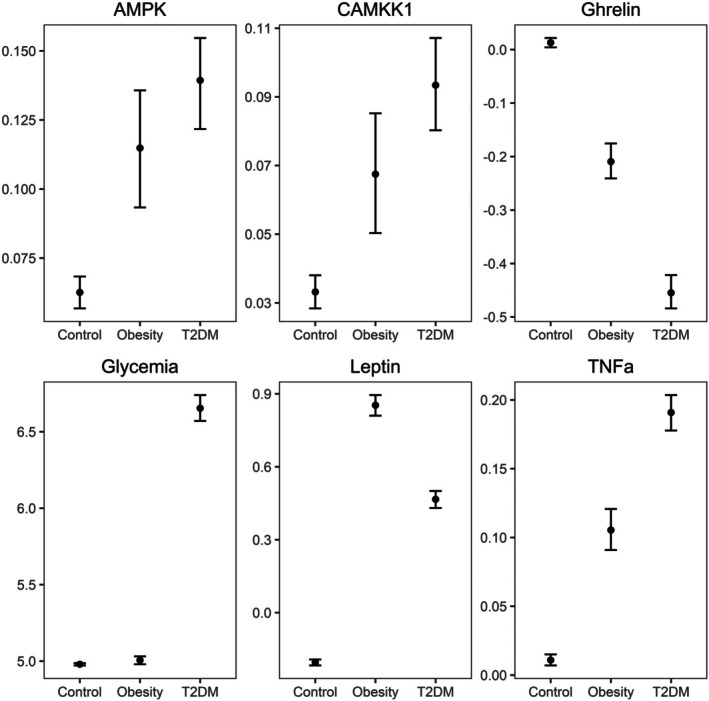
Mean serum levels across controls, individuals with obesity and participants with type 2 diabetes mellitus (T2DM). Whiskers represent confidence intervals.

Patients with T2DM had higher CAMKK1 in comparison to both individuals with obesity and controls (Figure 1, Table 1). However, in contrast to differences between individuals with T2DM and controls, differences in CAMKK1 serum levels between individuals with obesity and controls (age and sex‐adjusted ANCOVA, effect of diagnosis, *F* = 28.805, *p* < 0.001, post hoc comparison Tukey‐adjusted *p* = 0.001) were not significant after accounting for BMI (age, sex and BMI‐adjusted ANCOVA, Tukey‐adjusted *p* = 0.292, Table 1).

Individuals with obesity had lower ghrelin, higher glycemia and higher leptin in comparison to controls (Figure 1, Table 1). Patients with T2DM exhibited lower ghrelin, higher leptin, higher AMPK, higher glycemia and higher TNFα in comparison to controls (Figure 1, Table 1). In turn, individuals with obesity exhibited lower CAMKK1, lower glycemia, lower TNFα and higher ghrelin and leptin in comparison to patients with T2DM (Figure 1, Table 1).

### 3.2 Correlates

Overall, AMPK (effect size by Fisher's *z* = 0.546), TNFα (Fisher's *z* = 0.144), blood glucose (Fisher's *z* = 0.016) and leptin (Fisher's *z* = 0.030) were positively correlated with CAMKK1. See Figure S1 for a graphical representation of results and further details on correlations between cytokines.

Multivariable regression confirmed a significant association between CAMKK1 and AMPK, as well as between CAMKK1 and TNFα, within all diagnostic groups (Table 2). By contrast, leptin and ghrelin were significantly associated with CAMKK1 only within controls (Table 2). Further analyses were aimed at investigating the hypothesis that individuals in different diagnostic groups may exhibit different relationships between CAMKK1 and other appetite‐regulating, metabolic or inflammatory factors.

**TABLE 2:** Associations between CAMKK1 and other serum biomarkers.

	Controls	Obesity	T2DM
*R* ^2^	0.251	0.278	0.273
Δ*R* ^2^ baseline model	+0.245*** *(p* < 0.001)	+0.271*** *(p* < 0.001)	+0.270*** *(p* < 0.001)
Leptin	*β* 0.010** (*p* 0.002)	*β* −0.002 (*p* 0.896)	*β* 0.012 (*p* 0.226)
Ghrelin	*β* 0.005* (*p* 0.048)	*β* 0.007 (*p* 0.476)	*β* −0.006 (*p* 0.421)
AMPK	*β* 0.400*** (*p* < 0.001)	*β* 0.426*** (*p* < 0.001)	*β* 0.420*** (*p* < 0.001)
Glycemia	*β* 0.001 (*p* 0.919)	*β* 0.004 (*p* 0.756)	*β* −0.002 (*p* 0.322)
TNFα	*β* 0.092*** (*p* < 0.001)	*β* 0.070*** (*p* < 0.001)	*β* 0.083*** (*p* < 0.001)

*Note:* Each cell reports the regression coefficient, by age, sex and BMI‐adjusted linear regression. Independent variable on the left, dependent variables by column. Δ*R* ^2^ baseline model = incremental variance explained beyond age, sex and BMI. Relative *p*‐value in parenthesis by model comparison (ANOVA).

Abbreviations: BMI, body mass index; T2DM, type 2 diabetes.

*** *p* < 0.001.

** *p* < 0.01.

* *p* < 0.5.

The positive association between CAMKK1 and leptin was attenuated in individuals with obesity in comparison to controls (Table 3), but no similar effect was detected in patients with T2DM (Table 3). By contrast, the positive association between CAMKK1 and TNFα was strengthened in patients with T2DM, but not individuals with obesity (Table 3). In contrast to analyses performed within‐groups (Table 2), the positive association between CAMKK1 and blood glucose here reached statistical significance but was observed only in patients with T2DM (i.e., neither controls, nor individuals with obesity; Table 3). The association between CAMKK1 and ghrelin did not reach statistical significance when computing moderating effects for diagnostic groups (Table 3). The positive association between CAMKK1 and AMPK was neither attenuated nor strengthened in different diagnostic groups (Table 3).

**TABLE 3:** Moderation effect, diagnostic groups.

	CAMKK1	CAMKK1:Obesity	CAMKK1:T2DM
Leptin	*β* 0.082*** (*p* < 0.001)	*β* −0.112*** (*p* < 0.001)	*β* 0.003 (*p* 0.895)
Ghrelin	*β* −0.065 (*p* 0.615)	*β* 0.026 (*p* 0.480)	*β* 0.036 (*p* 0.214)
AMPK	*β* 0.598*** (*p* < 0.001)	*β* 0.031 (*p* 0.132)	*β* 0.019 (*p* 0.249)
Glycemia	*β* 0.017 (*p* 0.154)	*β* 0.014 (*p* 0.771)	*β* 0.105** (*p* 0.006)
TNFα	*β* 0.118*** (*p* < 0.001)	*β* 0.004 (*p* 0.797)	*β* 0.030* (*p* 0.023)

*Note:* Each cell reports the regression coefficient, by age, sex and BMI‐adjusted linear regression. Dependent variable on the left, independent variables by column.

Abbreviations: BMI, body mass index; CAMKK1:Obesity, interaction term (contrast with controls); CAMKK1:T2DM, interaction term (contrast with controls); T2DM, type 2 diabetes.

*** *p* < 0.001.

** *p* < 0.01.

* *p* < 0.5.

In patients with T2DM, CAMKK1 was not significantly associated with glycated haemoglobin (*β* = −0.002, *p* = 0.678). Being treated with insulin products (*F* = 0.353, post hoc comparison Tukey‐adjusted *p* = 0.552), metformin (*F* = 0.027, post hoc comparison Tukey‐adjusted *p* = 0.871), or both (*F* = 0.053, post hoc comparison Tukey‐adjusted *p* = 0.819) was not associated with different serum levels of CAMKK1. However, higher CAMKK1 was significantly associated with having complications due to diabetes (standardised *β* = 1.060, *p* = 0.010), and this effect was stronger in older individuals (standardised *β* = 0.041, *p* = 0.005) as well as in individuals with higher BMI (standardised *β* = 0.015, *p* = 0.029). The association between CAMKK1 and diabetic complications was not different between males and females (standardised *β* = 0.223, *p* = 0.762).

### 3.3 Network Analysis

Three different networks were estimated, one for each diagnostic group. Comparisons after bootstrapping (*N* = 5,000) confirmed that the networks significantly differed both in terms of structure (*p* < 0.001) and strength (*p* < 0.001) when comparing controls to patients with T2DM, but not to individuals with obesity (*p* = 0.516 and *p* = 0.626, for structure and strength, respectively). Moreover, network structure (*p* = 0.004) and strength (*p* < 0.001) differed between individuals with obesity and patients with T2DM.

**FIGURE 2:**
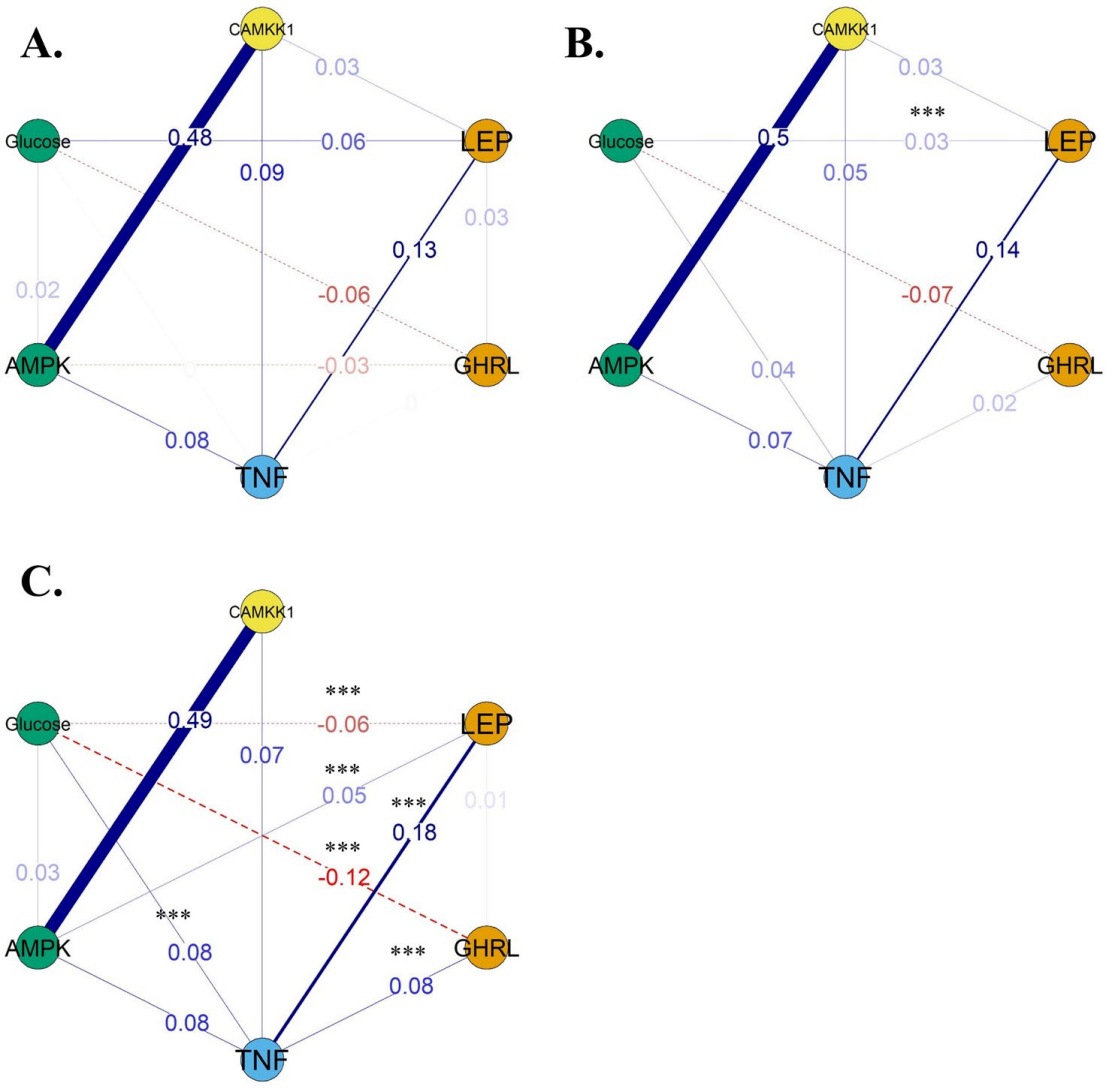
Network structure of CAMKK1, appetite‐regulating, metabolic and inflammatory factors. Each node represents the serum levels of biomarkers. Edges represent the pairwise association between the two proteins and between a protein and glucose levels. Network structure and edge strength estimated by mixed graphical models (10‐fold cross‐validated). Differences between networks were computed after bootstrapping (*N* = 5000). Asterisks in panel (B) = significant differences between individuals with obesity and patients with T2DM; asterisks in panel (C) = significant differences between patients with T2DM and controls. No asterisks reported in panel A as no significant differences were observed between controls and individuals with obesity. Panels: (A) Controls; (B) individuals with obesity; (C) patients with Type 2 diabetes. ***p < 0.001.

In particular, the association between leptin and TNFα was stronger in patients with T2DM (FDR *p* = 0.015), and an association between ghrelin and TNFα was observed in patients with T2DM but not controls (FDR *p* < 0.001). Similarly, the association between leptin and AMPK was observed in patients with T2DM but not controls (FDR *p* 0.015), as the association between TNFα and glucose (FDR *p* < 0.001). The association between leptin and glucose was positive in controls, positive but reduced in patients with obesity (FDR *p* = 0.006) and negative in patients with T2DM (FDR *p* < 0.001). The negative association between ghrelin and glucose was stronger in patients with T2DM in comparison to controls (FDR *p* = 0.014). An association between CAMKK1 and leptin was observed in both controls and individuals with obesity, but not in patients with T2DM. However, this lack of association did not survive correction for multiple comparisons (FDR *p* = 0.532). See Figure 2 for a graphical representation of results.

## 4 Discussion

The present work offers compelling evidence that CAMKK1 serum levels are increased in patients with T2DM, in comparison to both individuals with obesity and controls. CAMKK1 showed evidence of both direct and indirect associations with appetite‐regulating (i.e., leptin and ghrelin), metabolic (i.e., AMPK and glycemia) and inflammatory factors (i.e., TNFα). CAMKK1 associations with appetite‐regulating, metabolic and inflammatory factors were perturbed by T2DM, with preliminary evidence of diverging patterns of alterations within both obesity and T2DM (i.e., attenuated satiety signalling for obesity; strengthened inflammatory elevation for T2DM). In brief, current results suggest that blood glucose control may shift from pro‐homeostatic mechanisms to inflammatory control in patients with T2DM, with partial alterations already observable within patients with obesity alone.

As previously mentioned, current results show elevated CAMKK1 in patients with T2DM, suggesting that CAMKK1 may be a biomarker capable of differentiating between individuals with obesity and patients with T2DM. In fact, differences in CAMKK1 serum levels between individuals with obesity and controls were not significant after accounting for BMI, indicating that CAMKK1 serum levels were not further elevated beyond what explained by BMI alone in this population. By contrast, CAMKK1 levels were significantly different between patients with T2DM and controls even after accounting for BMI. Previous works investigated the role of CAMKs, in particular CAMKII, in the determination of maladaptive structural and functional changes in T2DM, namely in the vascular epithelium [38], cardiac myocytes, skeletal muscle cells [39] and adipocytes [40]. Future studies may then investigate whether these CAMKs‐induced structural and functional maladaptive changes may exhibit divergent dynamics in individuals with obesity or patients with T2DM, possibly accounting for effects beyond BMI alone.

Current results also corroborate previous studies on AMPK disruption in T2DM [41]. Previous studies described AMPK disruption in T2DM as possibly implicating CAMKK2 as an upstream druggable target for this clinical condition [42, 43]. However, to the best of the authors' knowledge, no previous study has described either CAMKs or CAMKK1 serum levels in either individuals with obesity or patients with T2DM. Nonetheless, previous works in animal models suggest that both CAMKs and CAMKK1 are localised in pancreatic *β*‐cells and their cell lines, and the exposure to glucose increases their expression [8]. Future studies may thus be interested in better characterising CAMKK1's role in T2DM, as well as the direction of causality between increased CAMKK1's expression and elevated blood glucose.

In this perspective, current results confirm the expected positive association between CAMKK1 and AMPK serum levels, as AMPK is a known direct downstream target of both CAMKK1 and CAMKK2 [17]. Moreover, current results indicate CAMKK1 as positively associated with leptin. This result may suggest that CAMKs and CAMKK1 contribute to a previously described signal transduction pathway, namely that linking leptin serum levels with intracellular AMPK activation [44]. This result may also be interpreted considering that CAMKK1 activates CREB transcription factors, and thus that elevated CAMKK1 may elevate leptin serum levels under the effect of CREB [45, 46]. Additionally, the positive association between CAMKK1 and TNFα may be interpreted in light of previous evidence, which linked CAMKK1 expression to increased functional activation and differentiation of circulating neutrophils [22]. In fact, neutrophils are one of the main cellular sites of production for TNFα during infections, generalised inflammation or wound healing [47, 48, 49].

Moderation analyses indicated that CAMKK1 may exhibit different associations with appetite regulating, metabolic and inflammatory factors according to diagnostic groups. In particular, the direct association between CAMKK1 and leptin was observed as attenuated in individuals with obesity, while the direct association between CAMKK1 and blood glucose or TNFα was strengthened in patients with T2DM. Taken together, these findings, as previously mentioned, suggest that CAMKK1's role may shift from pro‐homeostatic mechanisms (i.e., promoting ghrelin‐mediated food intake on one hand and promoting leptin‐mediated satiety on the other) to inflammatory control (i.e., TNFα‐mediated metabolic dysregulation) in patients with T2DM.

The attenuation of the relationship between CAMKK1 and leptin in individuals with obesity may also suggest that other metabolic factors may be involved in leptin‐binding cascades. One hypothesis might include other proteins within the intracellular AMPK downstream pathway [44], or the elevation of leptin by the previously mentioned activation of CREB transcription cascades [45, 46]. Both may then lead to an attenuation of pro‐satiety signalling in individuals with obesity. Future molecular studies are needed to replicate these results, and future studies are also needed to better characterise the crosstalk between CAMKK1 and leptin, as well as the potential interplay with other metabolic pathways. Future studies might also explore whether CAMKK1 or its downstream factors may exhibit allosteric states or secondary effector modulators. Such evidence would provide a mechanistic basis for current findings, which showed a strengthened association between CAMKK1 and blood glucose or TNFα in patients with T2DM.

Overall, current findings offer promising future potential for translational medicine. In fact, insulin products or metformin were here not observed as associated with different serum levels of CAMKK1, suggesting that these pharmacological products may not exert their mechanism of action through CAMKK1's modulation. However, elevated CAMKK1 was significantly associated with having complications due to diabetes, and this effect was stronger in older individuals and individuals at higher BMI, suggesting that current pharmacological products may not fully protect against potential complications of diabetes arising due to CAMKK1 elevation. However, future longitudinal and mechanistic studies are needed to confirm the hypothesis that CAMKK1 elevations are significantly and causally associated with complications due to diabetes in patients with T2DM.

Overall, CAMKK1's associations with other appetite‐regulating, metabolic and inflammatory factors here appeared to mostly follow a dose–response relationship, with no opposite directions of effects between diagnostic groups, and thus minimal hormetic effects. This result is particularly interesting for future translational research as this potential mechanism of action does not seem to be leveraged by current drugs, while also considering that developing analogues, agonists or antagonists for hormetic hormones often results in ineffective treatments [50], or in treatments with a high rate of side effects [51], due to the complex mechanisms underlying their effects at different doses, in different conditions, across different tissues and different molecular targets [52]. In this perspective, future research may validate CAMKK1 and other CAMKs as molecular targets for the treatment of either obesity or T2DM.

Future studies might also investigate the interplay between CAMKs and the neuropsychological dimensions of hunger, appetite and reward. In fact, CAMKs have been described as interacting with the serotonergic [45, 46], dopaminergic [53, 54] and cannabinergic systems [55], with significant implications for the treatment of obesity and diabetes. Interestingly, preliminary evidence suggests that restoring CAMKs expression might counteract at least part of the detrimental effects of diabetes on the central nervous system, promoting neuroplasticity, as evaluated in animal models [56]. CAMKs' role in neuroplasticity may also explain previous evidence, which described CAMKs signalling as crucial for mediating depression‐related behaviours [57]. However, future studies are needed to investigate the translational potential of these previous animal studies in humans.

### 4.1 Limitations

Present results offer persuading evidence for elevated CAMKK1 levels in both individuals with obesity and patients with T2DM in comparison to controls. Nonetheless, some limitations might hinder the generalisability of current findings. First, the UK Biobank cohort is predominantly composed of middle‐aged and older adults, which may restrict the generalisability of the findings to younger populations. Moreover, given the cross‐sectional design of our study, causal inference for present results could not be drawn. An important consideration is thus whether the observed increase in serum CAMKK1 represents a causal factor in the pathophysiology of T2DM or a secondary consequence of metabolic disease. Therefore, while our findings highlight a robust association between CAMKK1 levels and T2DM status, longitudinal and mechanistic studies will be essential to establish whether CAMKK1 plays an etiological role or reflects downstream alterations in energy and glucose metabolism, as well as whether CAMKK1 elevation may be described as either adaptive or maladaptive in nature. Detailed information on diabetes duration, treatment regimens and C‐peptide concentrations was not systematically available for the entire cohort. This limitation precluded more refined subgroup analyses and further exploration of potential clinical and molecular mechanisms underlying the observed associations, which were here not fully described. Finally, the UK Biobank, while offering extensive sample size, has been previously characterised as exhibiting reduced ethnic and racial diversity. Future studies might explore the generalisability of current findings across different ethnic and racial backgrounds.

## 5 Conclusions

The present work shows how CAMKK1 expression is elevated in both individuals with obesity and patients with T2DM, in comparison to controls. However, the difference between individuals with obesity and controls was not significant after accounting for BMI. CAMKK1 showed evidence of direct and indirect associations with appetite‐regulating (i.e., leptin and ghrelin), metabolic (i.e., AMPK and glycemia) and inflammatory factors (i.e., TNFα). These associations were observed as perturbated by both obesity and T2DM, showing how physiological signalling may be disrupted in these conditions. Taken together, current results including preliminary evidence of clinical associations between elevated CAMKK1 and complications due to diabetes, show how blood glucose control may be described as shifting from pro‐homeostatic mechanisms (i.e., satiety and hunger, leptin and ghrelin) to inflammatory control (i.e., TNFα) in T2DM.

## Author Contributions

L.T.: conceptualisation, methodology, formal analysis, investigation, data curation, writing – original draft, writing – review and editing, visualisation. L.B.: conceptualisation, methodology, investigation, data curation, writing – review and editing. A.D.S.: conceptualisation, investigation, writing – review and editing. P.R.: conceptualisation, investigation, writing – review and editing, supervision, project administration. C.S.: conceptualisation, investigation, writing – review and editing, supervision, project administration. R.G.: conceptualisation, investigation, writing – review and editing. R.S.: conceptualisation, investigation, writing – review and editing, supervision, project administration. G.V.: conceptualisation, investigation, writing – review and editing, supervision, project administration. R.N.: conceptualisation, investigation, writing – review and editing, resources, supervision, project administration. F.D.L.: conceptualisation, investigation, writing – review and editing, resources, supervision, project administration. V.R.: conceptualisation, investigation, supervision, funding acquisition, writing – review and editing, project administration. G.C.: conceptualisation, investigation, validation, supervision, funding acquisition, writing – original draft, writing – review and editing, project administration.

## Ethics Statement

The UK Biobank is a prospective cohort study with a large catalogue of phenotypic and genotypic data from ~500,000 individuals. Participants were residents of the UK and represented a range of sociodemographic backgrounds, providing a comprehensive sample of the adult to elderly population. The study was approved by the North West Centre for Research Ethics Committee (11/NW/0382), and the participants provided signed consent before examination. See the parent study for further information [27].

## Conflicts of Interest

The authors declare no conflicts of interest.

## Supporting information


**Table S1:** Post hoc comparisons, CAMKK1.
**Figure S1:** Correlation structure of serum levels of CAMKK1, appetite‐regulating, metabolic and inflammatory factors, as measured in controls, individuals with obesity and participants with type 2 diabetes mellitus. Each cell represents the pairwise correlation (Pearson's r, age and BMI‐adjusted). Individuals were removed row‐wise in case of missing values. Minimum sample size, *n* = 43,440. ***p < 0.001, **p < 0.01, *p < 0.5.

## Data Availability

The data that support the findings of this study are available from UKBioBank. Restrictions apply to the availability of these data, which were used under licence for this study. Data are available from https://www.ukbiobank.ac.uk/ with the permission of UKBioBank. This research has been conducted using the UK Biobank Resource under Application Number 501518
