# Supplementary material for: CAMKK1 in Obesity and Type 2 Diabetes Mellitus: Evidence of Interaction With Appetite‐Regulating, Metabolic and Inflammatory Factors
Source: Endocrinol Diabetes Metab. 2025 Sep 18;8(5):e70109. doi: 10.1002/edm2.70109 (PMC12445118; doi:10.1002/edm2.70109)
Supplement: Supplementary file 1 — Table S1: Post hoc comparisons, CAMKK1. Figure S1: Correlation structure of serum levels of CAMKK1, appetite‐regulating, metabolic and inflammatory factors, as measured in controls, individuals with obesity and participants with type 2 diabetes mellitus. Each cell represents the pairwise correlation (Pearson's r, age and BMI‐adjusted). Individuals were removed row‐wise in case of missing values. Minimum sample size, n = 43,440. ***p < 0.001, **p < 0.01, *p < 0.5. [file EDM2-8-e70109-s001.docx]

**Supplementary Materials**

| **Table S1 - Post-hoc comparisons, CAMKK1** | | | | | |
| --- | --- | --- | --- | --- | --- |
| **Group** | **Comparison** | **Mean Difference** | **SE** | **T-value** | **p_tukey_** |
| Female Control | Male Control | -0.020 | 0.005 | -4.061 | < .001 |
|  | Female Obesity | -0.057 | 0.013 | -4.543 | < .001 |
|  | Male Obesity | -0.022 | 0.015 | -1.481 | 0.676 |
|  | Female T2DM | -0.081 | 0.012 | -6.709 | < .001 |
|  | Male T2DM | -0.055 | 0.010 | -5.447 | < .001 |
| Male Control | Female Obesity | -0.037 | 0.013 | -2.948 | 0.038 |
|  | Male Obesity | -0.002 | 0.015 | -0.120 | 1.000 |
|  | Female T2DM | -0.061 | 0.012 | -5.020 | < .001 |
|  | Male T2DM | -0.035 | 0.010 | -3.440 | 0.008 |
| Female Obesity | Male Obesity | 0.036 | 0.019 | 1.909 | 0.397 |
|  | Female T2DM | -0.023 | 0.017 | -1.394 | 0.731 |
|  | Male T2DM | 0.002 | 0.015 | 0.156 | 1.000 |
| Male Obesity | Female T2DM | -0.059 | 0.018 | -3.231 | 0.016 |
|  | Male T2DM | -0.033 | 0.017 | -1.950 | 0.371 |
| Female T2DM | Male T2DM | 0.026 | 0.015 | 1.729 | 0.512 |
| *Note.* P-value adjusted for comparing a family of 6 estimates. | | | | | |


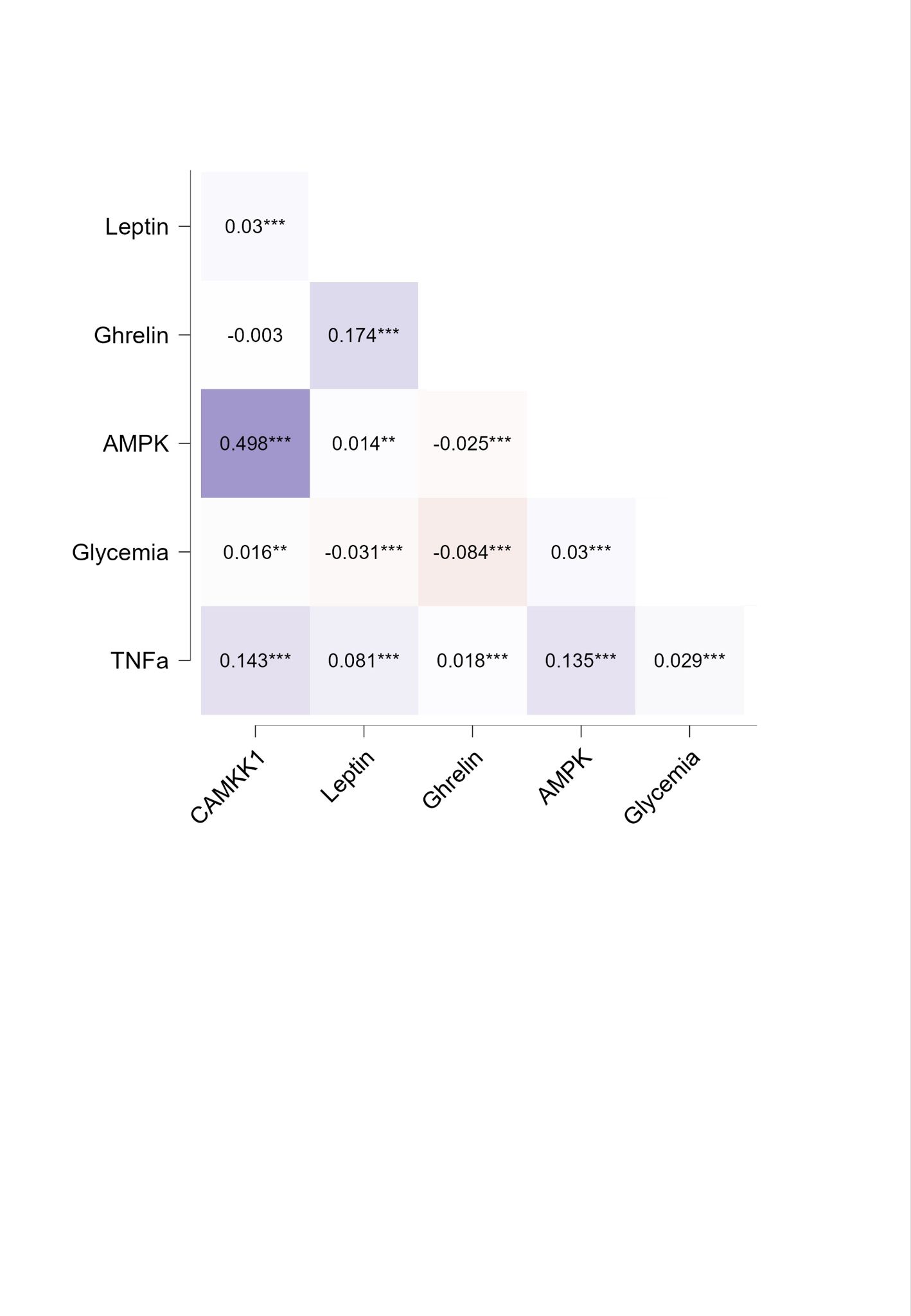


**Figure S1**  - Correlation structure of serum levels of CAMKK1, appetite-regulating, metabolic and inflammatory factors, as measured in controls, individuals with obesity and participants with type 2 diabetes mellitus. Each cell represents the pairwise correlation (Pearson’s r, age and BMI adjusted). Individuals were removed row-wise in case of missing values. Minimum sample size, n = 43,440.
*Legend:* *** p < 0.001, ** p < 0.01, * p < 0.5
